# Supplementary material for: Exploration of the Different Dimensions of Wurtzite ZnO Structure Nanomaterials as Gas Sensors at Room Temperature
Source: Nanomaterials (Basel). 2023 Oct 23;13(20):2810. doi: 10.3390/nano13202810 (PMC10609452; doi:10.3390/nano13202810)
Supplement: Supplementary file 1 [file nanomaterials-13-02810-s001.zip › nanomaterials-2633137-supplementary.pdf]

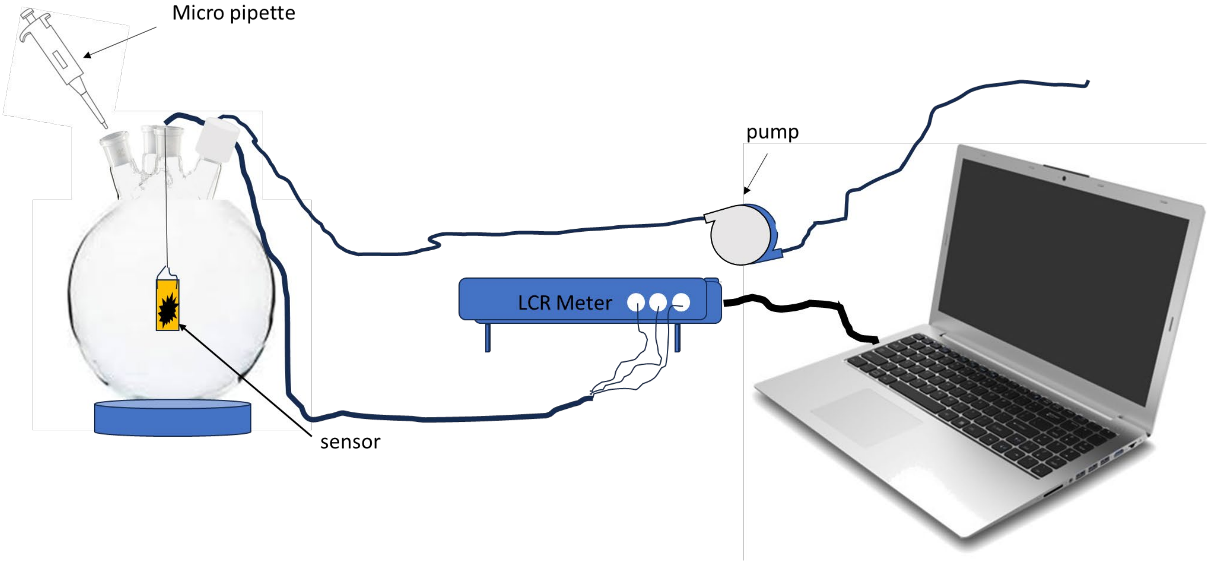

Figure S1. Sensors performance measurement setup.

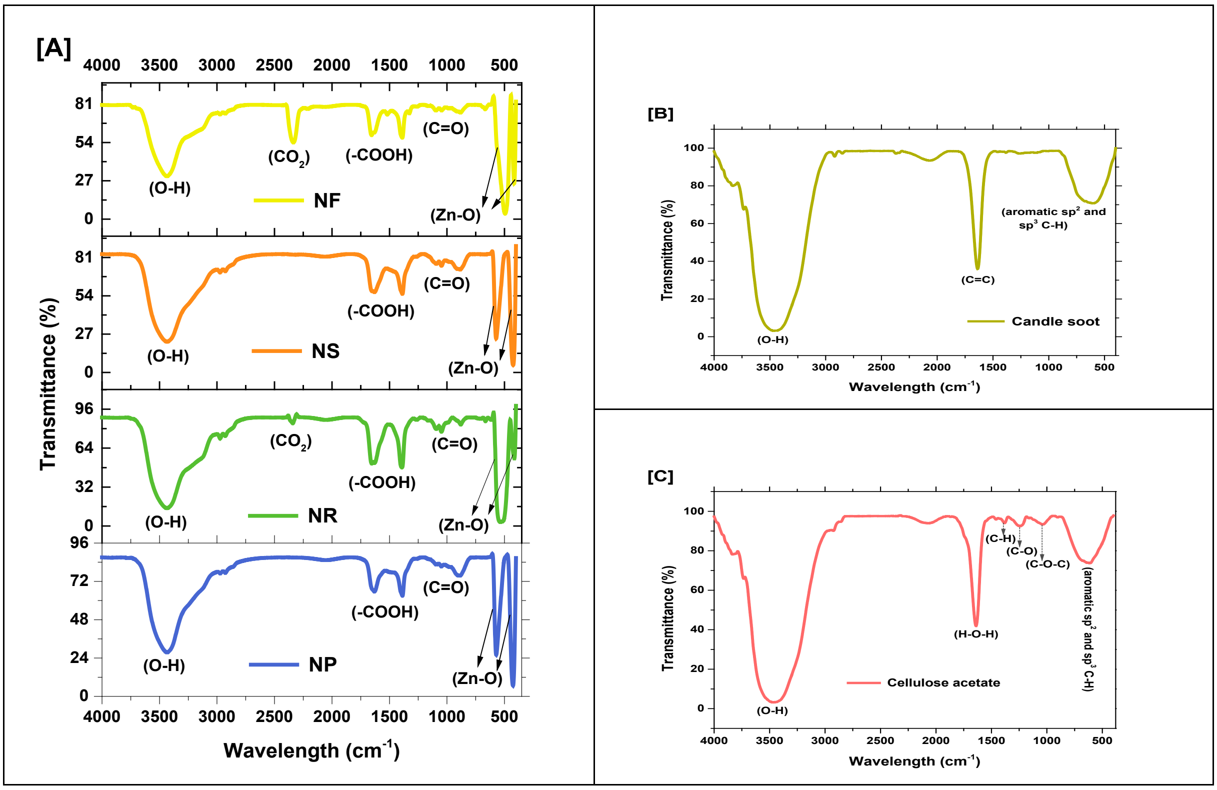

Figure S2. FTIR spectra of the (A) synthesized ZnO nanostructures, i.e., nanoflowers (N.F.), nanosheets (N.S.), nanorods (N.R.), and nanoparticles (N.P.); (B) candle soot, and (C) purchased cellulose acetate respectively.

BET isotherm

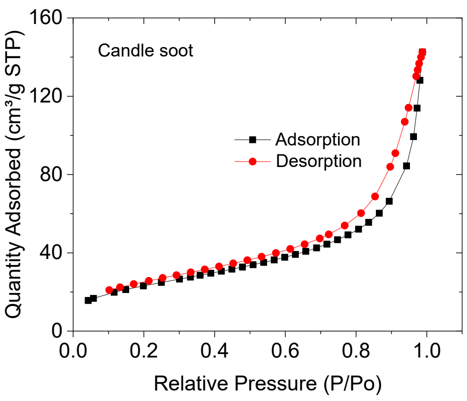

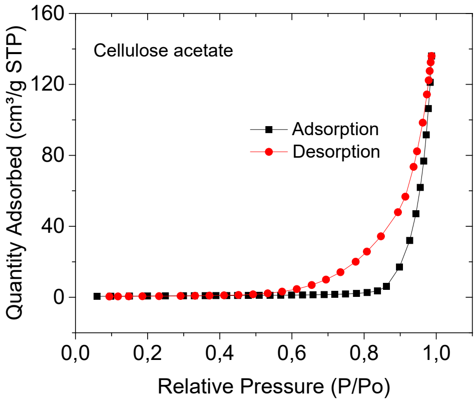

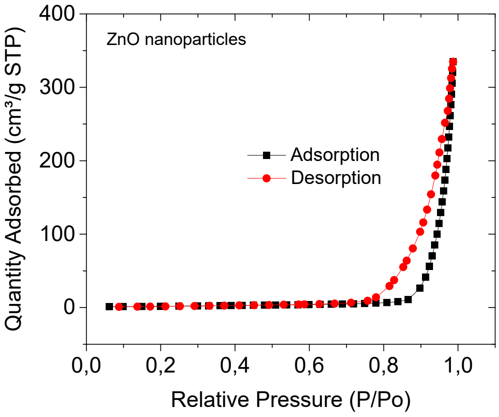
ZnO nanoparticles
Adsorption
Desorption
Quantity Adsorbed (cm³/g STP)
Relative Pressure (P/Po)

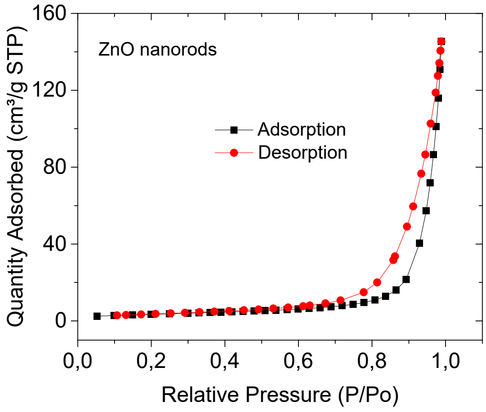
ZnO nanorods
Adsorption
Desorption
Quantity Adsorbed (cm³/g STP)
Relative Pressure (P/Po)

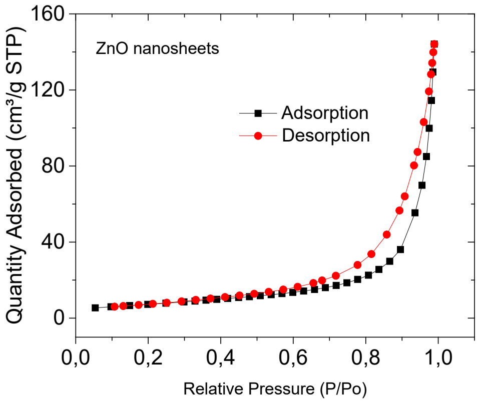
ZnO nanosheets
Adsorption
Desorption
Quantity Adsorbed (cm³/g STP)
Relative Pressure (P/Po)

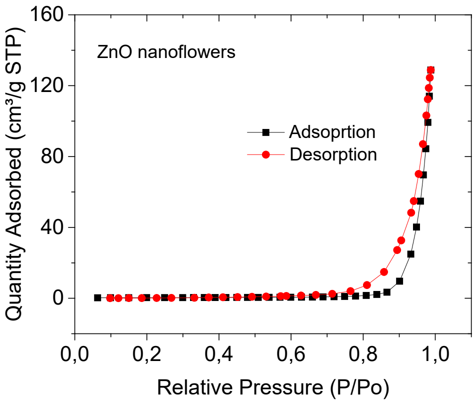
ZnO nanoflowers
Adsoprtion
Desorption
Quantity Adsorbed (cm³/g STP)
Relative Pressure (P/Po)

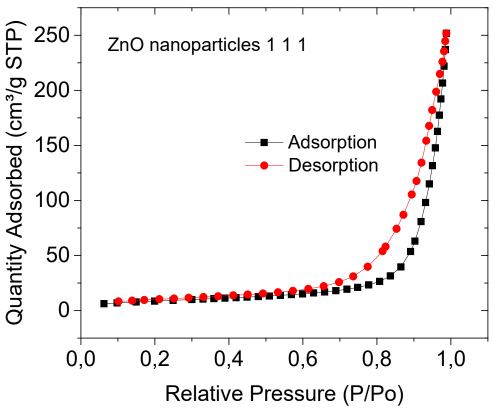
ZnO nanoparticles 1 1 1
Adsorption
Desorption
Quantity Adsorbed (cm³/g STP)
Relative Pressure (P/Po)

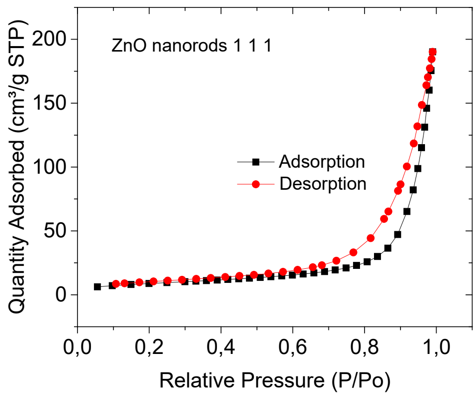
ZnO nanorods 1 1 1
Adsorption
Desorption
Quantity Adsorbed (cm³/g STP)
Relative Pressure (P/Po)

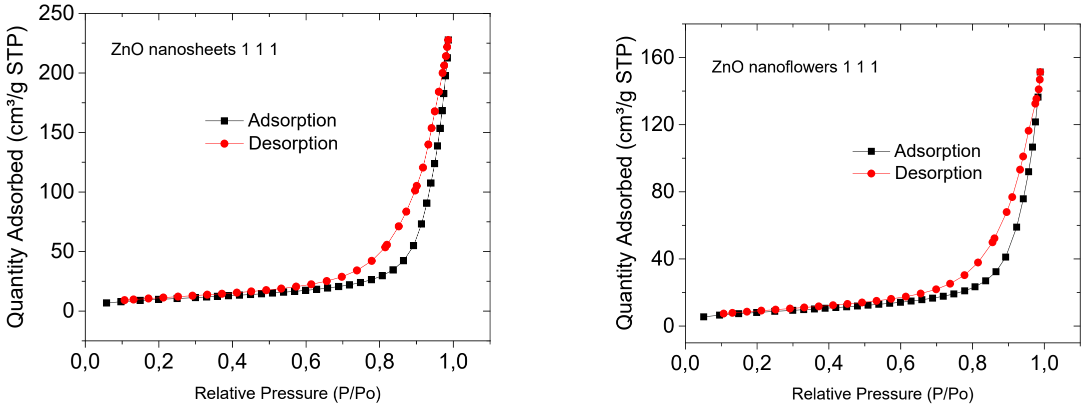

Figure S3. BET isotherm.

UV-vis diffuse reflectance

The UV-vis diffuse reflectance spectra and respective bandgap energies of the as-synthesized ZnO nanostructures are shown in Figure S3a and b, respectively. The diffuse reflectance spectra of the nanoparticles, nanorods, nanosheets and nanoflowers showed a sharp increase in reflectance at 378 nm, 374 nm, 371 nm, and 381 nm, respectively. The measured reflectance spectra were transformed to the corresponding absorption spectra by applying the Kubelka–Munk function (F(R) = (1 − R)2/2R) and plotted against the photon energy [1]. The bandgap energy was estimated from the x-axis intersection point from the extrapolation of the linear increase of light absorption with increasing energy, as displayed in Figure 4. The estimated band gap energy of the nanoparticles, nanorods, nanosheets and nanoflowers (see Table S1) corresponds to the violet-blue region of the electromagnetic spectrum [2]. The band gap of ZnO crystal at room temperature is 3.3 eV [3]. As expected, though, the band gap energies of the different morphologies of the ZnO nanostructures were not the same because band gap energy depends on the morphology and crystal size [4]. The band gap energies of the ZnO nanostructures show an increase with a decrease in crystal size (calculated from the XRD results). The results agree well with the reported values [5]. At the nanoscale, the overlapping energy levels spread to become more quantized, widening the band gap in the materials [4].

Table S1. Band gap energy (Eg), Average crystallite size (d) and surface area (A) of nanostructured oxides.

ZnO morphology	Bandgap (eV)	The average crystallite size (nm)	surface area (m^2g^{-1})
Nanoflower	3.20	44	1.3
Nanoparticles	3.27	35	6.7
Nanorods	3.29	41	12.7
Nanosheet	3.32	28	26.8

The UV-vis diffuse reflectance spectrum of the generated candle soot shows a broader reflectance band and a band gap energy of 2.92 eV (Figure S3). The diffuse reflectance spectrum of the purchased cellulose acetate demonstrates a sharp increase in reflectance at 227.8 nm, corresponding to an estimated band gap energy of 5.35 eV (Figure S3).

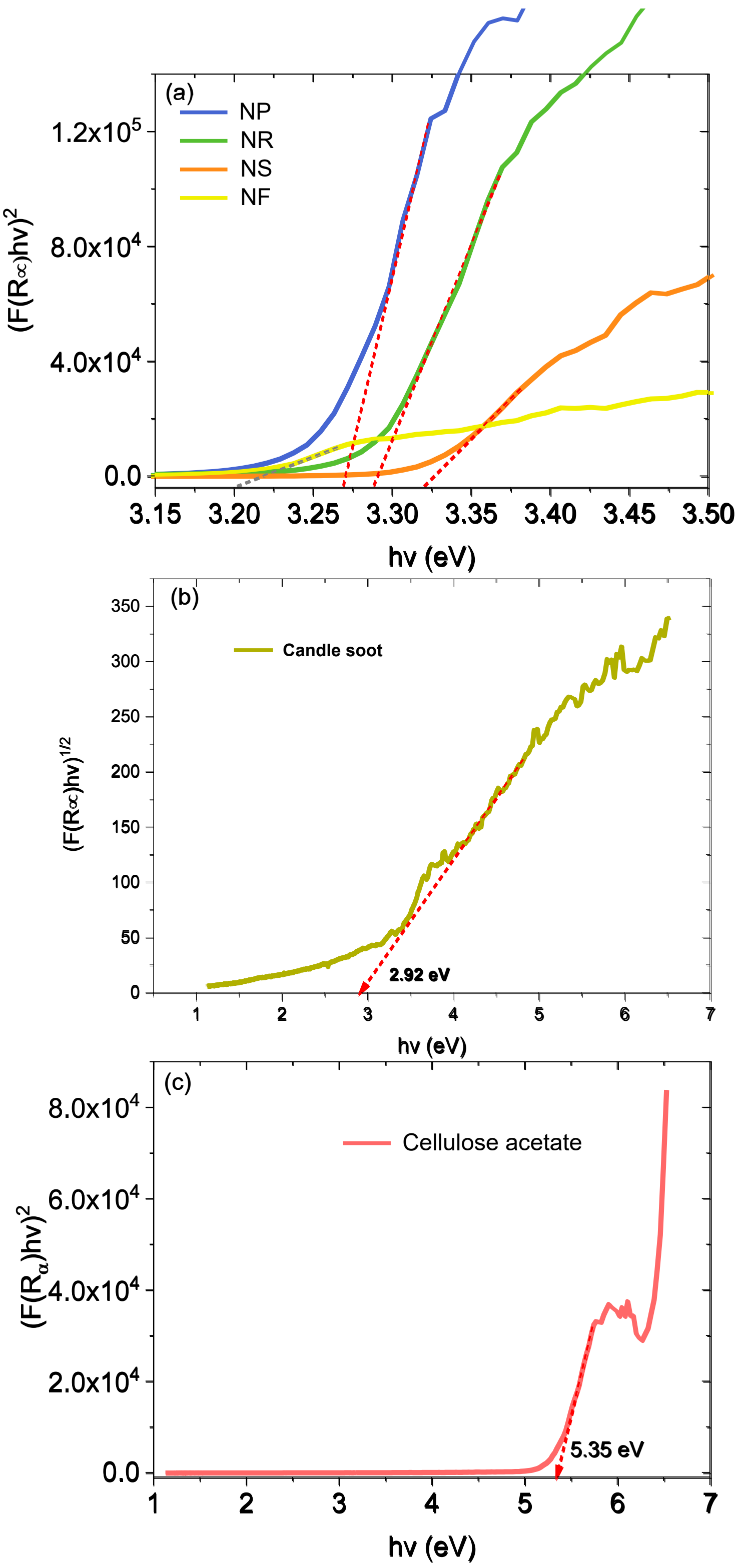

Figure S4. UV-vis diffuse reflectance spectra and bandgap energy values of the (a) synthesized ZnO nanostructures, i.e. nanoflowers (N.F.), nanosheets (N.S.), nanorods (N.R.), and nanoparticles (N.P.); (b) candle soot, and (c) purchased cellulose acetate.

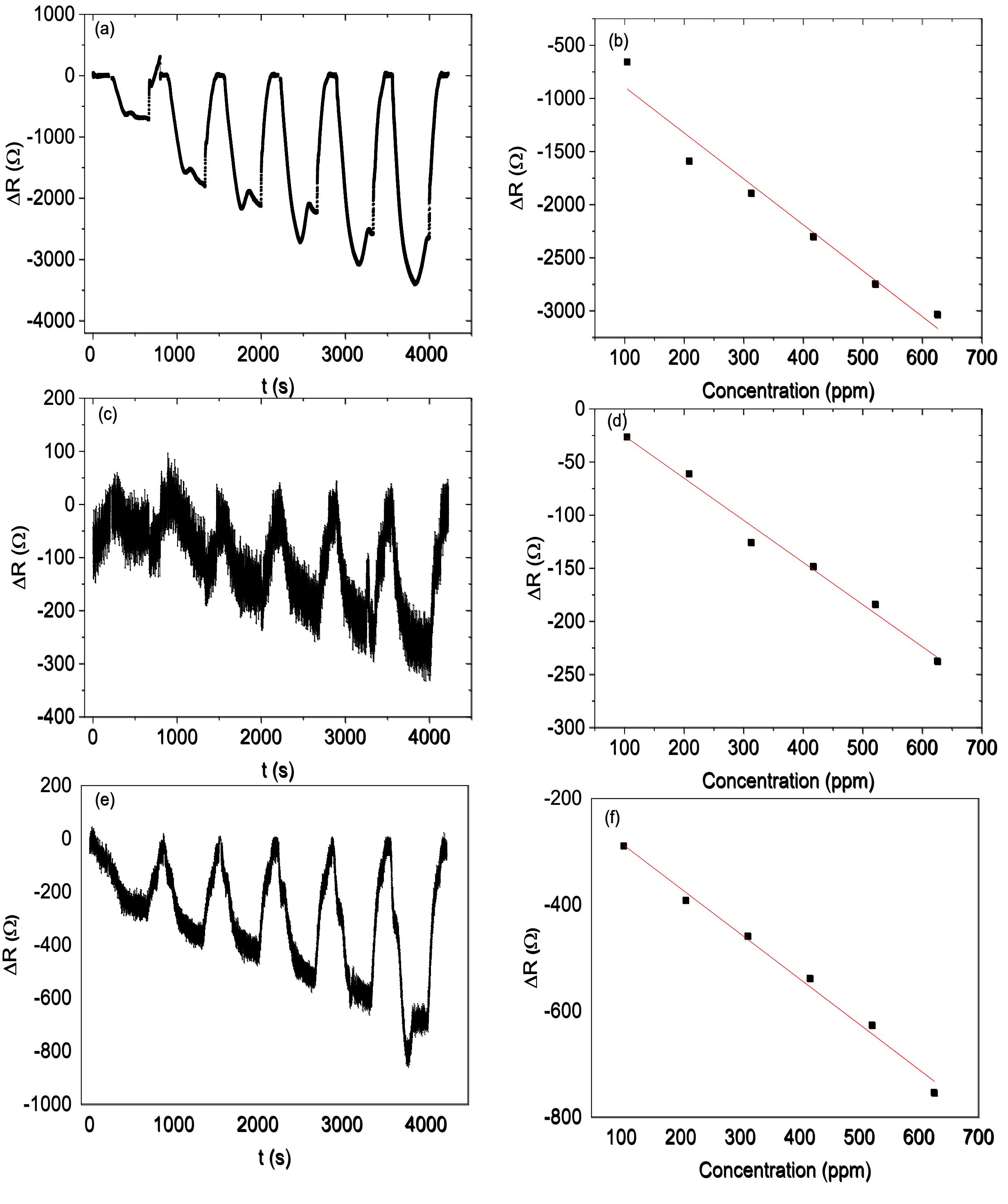

Figure S5. Dynamic response and recovery curves 3:1:1 mass ratio towards ethanol vapor (a) nanoparticles and (b) calibration curve; (c) nanorod and (d) its calibration curve; (e) nanoflower (f) its calibration curve;.

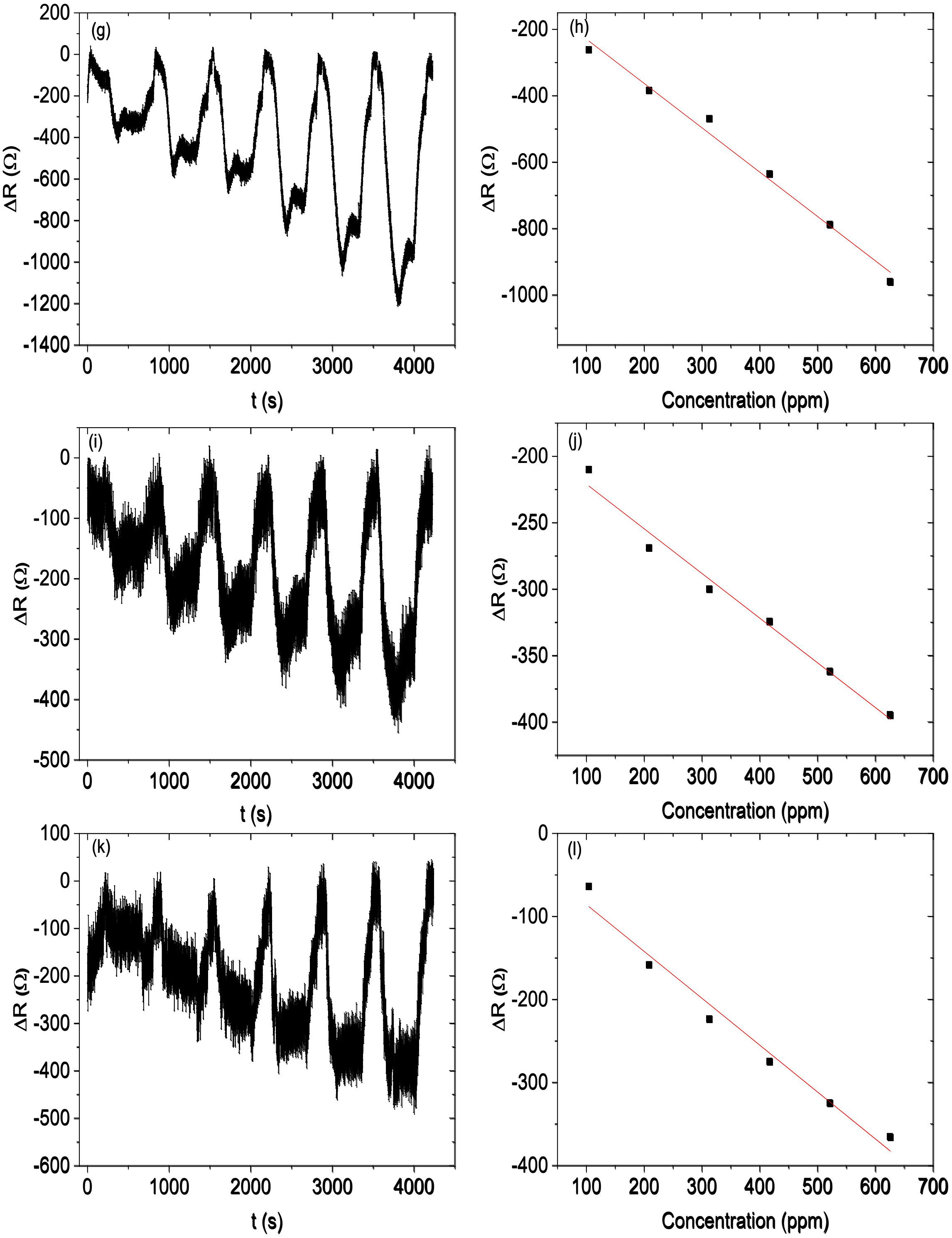

Figure S6. Dynamic response and recovery curves 2:1:1 mass ratio towards ethanol vapor (g) nanorod and (h) calibration curve; (i) nanosheet and (j) its calibration curve; (k) nanoflower (l) its calibration curve;.

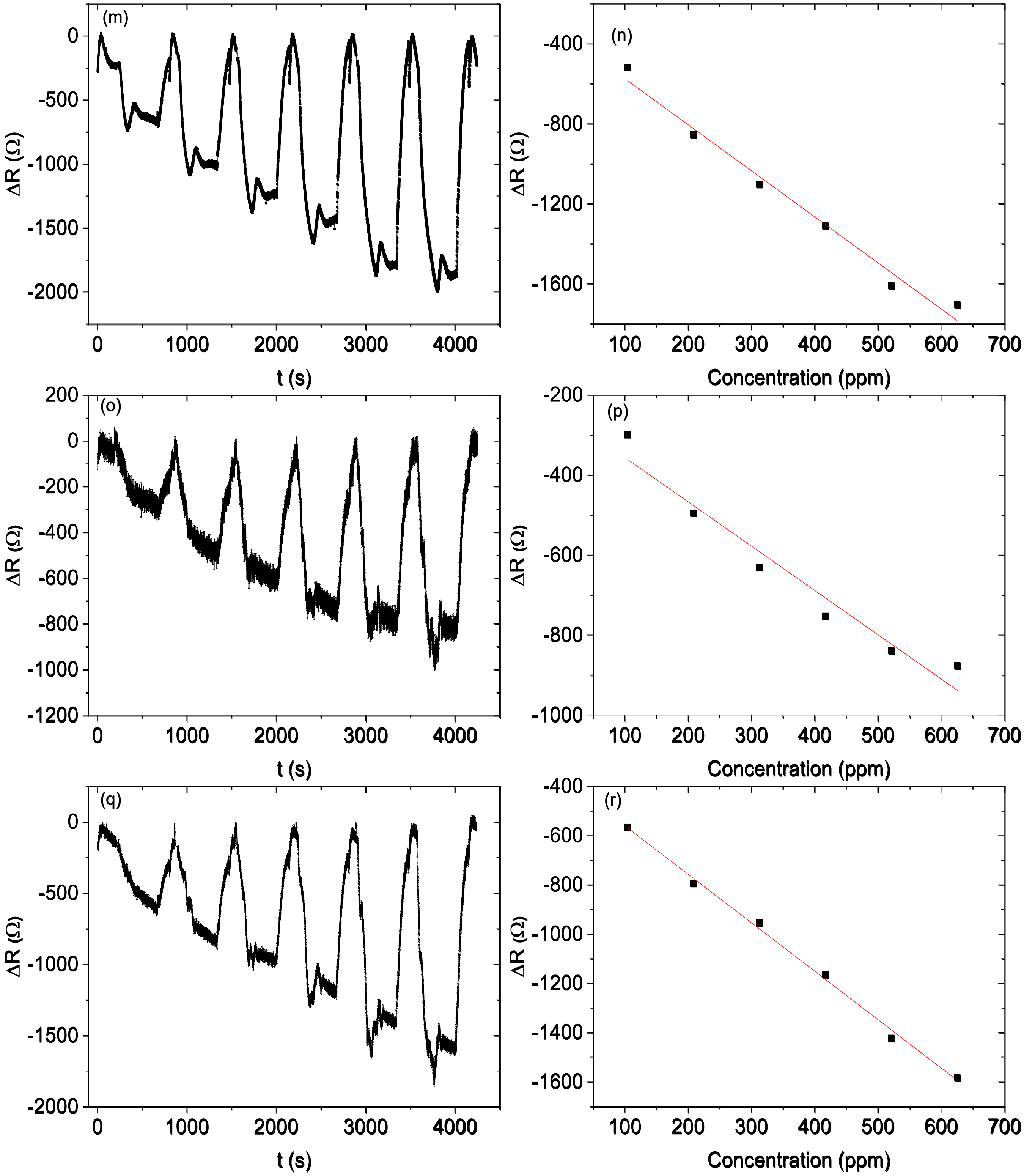

Figure S7. Dynamic response and recovery curves 1:1:1 mass ratio of towards isopropanol vapor(a) nanorod and (b) calibration curve; (c) nanosheet and (d) its calibration curve; (e) nanoflower (f) its calibration curve;.

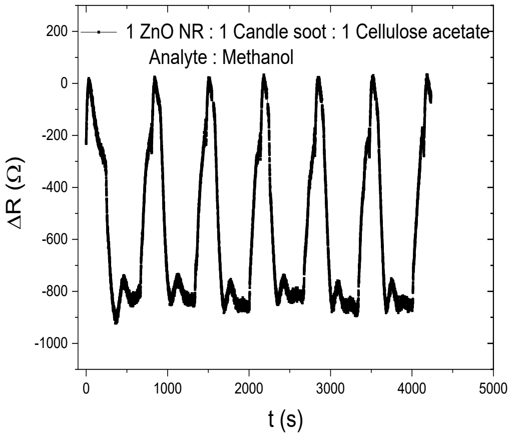

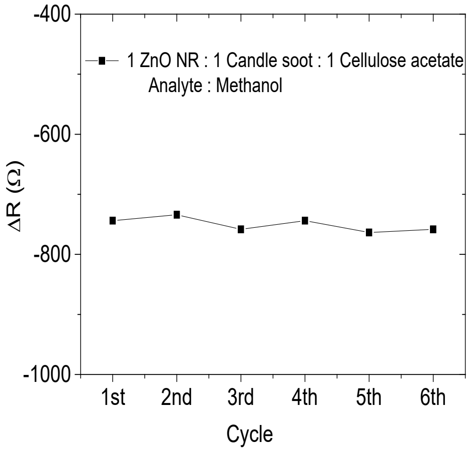

Figure S8. Static response and recovery curves 1:1:1 mass ratio of towards methanol.

Table S2. Summary of the performance of the fabricated sensors when detecting ethanol vapour.

**Sensor name**	**Sensitivity (Ω ppm^{-1})**	**Response time (min.)**	**Recovery time (min.)**
3:1:1-NP	4.3204	2.97	2.19
3:1:1-NR	0.3966	6.38	1.20
3:1:1-NS	0	0	0
3:1:1-NF	0.8520	2.41	2.81
2:1:1-NP	0	0	0
2:1:1-NR	1.3350	3.33	3.10
2:1:1-NS	0.3362	1.86	3.52
2:1:1-NF	0.5643	1.46	3.34
1:1:1-NP	0	0	0
1:1:1-NR	2.3007	2.92	2.09
1:1:1-NS	1.1067	2.08	3.35
1:1:1-NF	1.9670	3.50	3.39

Table S3. Summary of the performance of the fabricated sensors when detecting isopropanol vapor.

**Sensor name**	**Sensitivity (Ω ppm^{-1})**	**Response time (min.)**	**Recovery time (min.)**
3:1:1-NP	3.4539	1.73	2.25
3:1:1-NR	0	0	0
3:1:1-NS	0	0	0
3:1:1-NF	0	0	0
2:1:1-NP	0	0	0
2:1:1-NR	0.8077	2.38	2.72

2:1:1-NS	0.5070	2.48	1.45
2:1:1-NF	0	0	0
1:1:1-NP	0	0	0
1:1:1-NR	0	0	0
1:1:1-NS	0,8780	3.35	3.28
1:1:1-NF	2.4753	3.07	3.00

References

1. Ng, H. T., Chen, B., Li, J., Han, J., Meyyappan, M., Wu, J., S. Li, S., Haller, E.; Optical properties of single-crystalline ZnO nanowires on m-sapphire, Appl. Phys. Lett., 2003. 82(13): p. 2023-2025.
2. Yang, M. M., Crerar, D. A., Irish, D. E.; A raman spectroscopic study of lead and zinc acetate complexes in hydrothermal solutions, *Geochim. Cosmochim. Acta.*, 1989. 53(2): p. 319-326.
3. K. Zhang, K.; Feldner, A., Fischer, S.; FT Raman Spectroscopic Investigation of Cellulose Acetate, *Cellulose*, 2011. 18(4): p. 995-1003.
4. (a) Rusdi, R., Abd Rahman, A., Mohamed, N. S., Kamarudin, N., Kamarulzaman, N.; Preparation and band gap energies of ZnO nanotubes, nanorods and spherical nanostructures, *Powder Technol.*, 2011. 210(1): p. 18-22. (b) Makuła, P., Pacia, M., Macyk, W.; How To Correctly Determine the Band Gap Energy of Modified Semiconductor Photocatalysts Based on UV–Vis Spectra, *J. Phys. Chem. Lett*. 2018, 9, 23, 6814–6817.
5. Srikant V., Clarke, D.R.,; ZnO Films Deposited on Porous Silicon by DC Sputtering, *J. Appl. Phys.*, 1998. 83(10): p. 5447-5451.
